# Iconic Mathematics: Math Designed to Suit the Mind

**DOI:** 10.3389/fpsyg.2022.890362

**Published:** 2022-06-13

**Authors:** Peter Kramer

**Affiliations:** Department of General Psychology, University of Padua, Padua, Italy

**Keywords:** iconic mathematics, embodied mathematics, mathematical cognition, math education, stem education, dyscalculia, hypercalculia

## Abstract

Mathematics is a struggle for many. To make it more accessible, behavioral and educational scientists are redesigning how it is taught. To a similar end, a few rogue mathematicians and computer scientists are doing something more radical: they are redesigning mathematics itself, improving its ergonomic features. Charles Peirce, an important contributor to ordinary symbolic logic, also introduced a rigorous but non-symbolic, graphical alternative to it that is easier to picture. In the spirit of this *iconic logic*, George Spencer-Brown founded *iconic mathematics*. Performing iconic arithmetic, algebra, and even trigonometry, resembles doing calculations on an abacus, which is still popular in education today, has aided humanity for millennia, helps even when it is merely imagined, and ameliorates severe disability in basic computation. Interestingly, whereas some intellectually disabled individuals excel in very complex numerical tasks, others of normal intelligence fail even in very simple ones. A comparison of their wider psychological profiles suggests that iconic mathematics ought to suit the very people traditional mathematics leaves behind.

## Introduction: Toward a More Ergonomic Mathematics

In mathematics you don’t understand things, you get used to them.

*—attributed to John van Neumann* (Zukav, 1979)

### Icons Versus Symbols

Mathematics is a widely used and highly effective tool. Yet over the course of some 3,000 years, it has developed more or less organically rather than according to a carefully thought-through, preconceived plan (Kline, 1990). Today, professional mathematicians concern themselves with mathematical problems, rarely with revising the system in which they are expressed, and much less with improving its ergonomic features; those are matters for others to worry about. Yet what those others, including math teachers and behavioral and educational researchers, focus on is not redesigning mathematics but helping people, especially children, become better at it. Indeed, overhauling such an enormous, already well-entrenched system as that of mathematics may seem too daunting a task to take seriously. Consider, however, that its imposing edifice (its derived rules or theorems) is based on a relatively small foundation (its ground rules or axioms). These axioms are not particularly complicated, and a lot can be achieved by tinkering specifically with them.

The present analysis of the concept of iconic mathematics argues that there is an opportunity for the behavioral and educational sciences to contribute to this endeavor. The goal would not be to break new ground in mathematics—let us leave this to the mathematicians—but rather to apply to math what we know about the human mind and make math easier to learn and use, more ergonomic. The point of departure for this project is Spencer-Brown’s (1969) seminal adaptation of Charles Peirce’s *iconic logic* (Roberts, 1973; Kauffman, 2001; Shin, 2002) that became the cornerstone of *iconic mathematics* (Kauffman and Varela, 1980; James, 1993; Kauffman, 1995; Bricken, 2019a,b, 2021).

Unlike their traditional counterparts, iconic logic and iconic mathematics shun arbitrary squiggles that have only conventional meanings, like digits and plus, minus, and other abstract symbols. As much as possible, they are “postsymbolic” (Bricken, 2019a), and use icons instead. By definition, icons are more concrete than symbols, and often illustrate their own meaning, are their own mnemonic devices, and hint at their own intended use. Euler and Venn diagrams, which tellingly are still very popular tools in teaching logical and set theoretical relationships today (Trafimow, 2011; Reani et al., 2019), can be seen as precursors of the more versatile, more encompassing systems of iconic logic and iconic mathematics. Here, I limit myself to the latter and offer my take on a recent, particularly substantial contribution to iconic mathematics by computer scientist Bricken (2019a,b, 2021) and his student James (1993). In the process, I introduce an alternative notation to render the original one considerably more concise and even more ergonomic. The goal is to demonstrate that ordinary, symbolic mathematics need not be the only game in town; that, without sacrificing rigor, one can aspire to develop a more user-friendly kind of mathematics that can be used either as a stepping stone to learning ordinary, symbolic mathematics or as an alternative to it.

Iconic mathematics is, as much as possible, an “embodied” mathematics (Bricken, 2019a). To introduce this concept and set the stage, I therefore begin with a brief introduction of embodiment in mathematical cognition and mathematical education, and then, in the main conceptual analysis, show how embodiment permeates the nuts and bolts of iconic mathematics itself. I discuss iconic number representation and iconic addition and subtraction, then iconic multiplication, division, and taking the power or logarithm of numbers, and after that—to demonstrate iconic math’s potential—iconic imaginary numbers and their relationship with trigonometry. After the main analysis, I address why ordinary mathematics, curiously enough, is more difficult for some intelligent individuals than for others deemed intellectually disabled. I lay out how both talent in mathematics, and the near-total lack of it, are related to genetic conflict and patterns in mental disease. On the basis of this material and additional evidence obtained with the abacus, I argue that iconic mathematics promises to be of help especially to those who, with traditional mathematics, tend to struggle the most.

### Embodiment Versus Abstraction

Ordinary symbolic mathematics is highly abstract, but mounting evidence suggests that mental number representations and mathematical operations are *embodied*—that is, grounded in Lakoff and Núñez (2000), or at least shaped or affected by Winter and Yoshimi (2020), the sensory experiences our bodies provide to us (for reviews, see Fischer and Brugger, 2011; Fischer and Shaki, 2018; Soylu et al., 2018; Barrocas et al., 2020; see also especially Fischer et al., 2021; Glenberg, 2021) and other studies not covered by the reviews: Hilton, 2019; Proverbio and Carminati, 2019; van den Berg et al., 2021. For example, whereas Germans are accustomed to counting to ten using two hands, the Chinese manage the same with just one, and as if forced to mentally switch hands, Germans take longer than the Chinese to identify the smaller (or larger) of such numbers as 4 and 6 but not 2 and 4. Likewise, as if numerical distances were physical, numbers are distinguished faster if they are numerically far apart, like 1 and 9, than if they are close together, like 1 and 3 (Dehaene, 2011). And, as if numbers were mentally represented along a number line, in people reading from left to right, numbers are processed quicker if small and presented on the left, or large and presented on the right, rather than the converse (Dehaene, 2011; see for a related review Umiltà et al., 2009 and for related research papers and references therein Ashkenazi and Henik, 2010; Longo and Lourenco, 2010; Pia et al., 2010; Kramer et al., 2011; Thomas et al., 2017; Patro et al., 2018). Relatedly, addition can be pictured as rightward, and subtraction as leftward, movement along this line (Knops et al., 2009; Marghetis et al., 2014). Alternative kinds of the embodiment have been observed in mathematical cognition too (Winter and Yoshimi, 2020).

**FIGURE 1:**
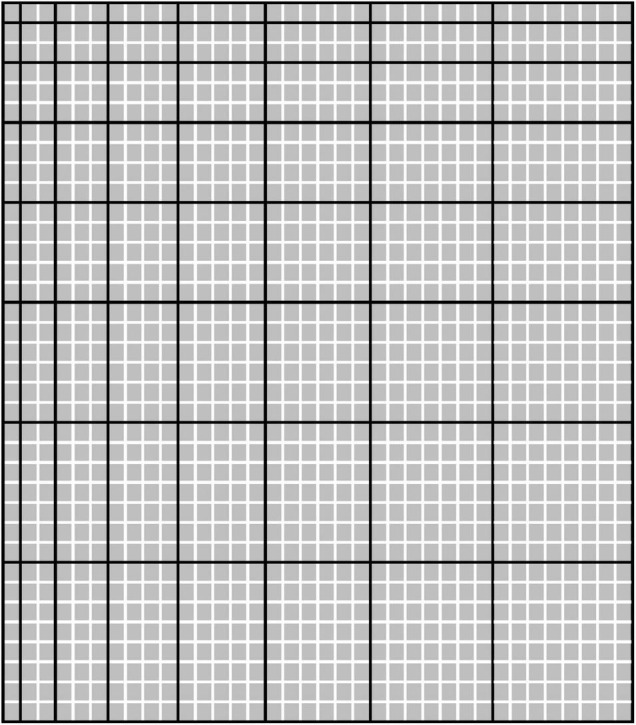
Laisant’s table [adapted from Maffia and Mariotti (2020)]. Each black-framed rectangle represents a multiplication—that of its width in little squares by its height in little squares. The outcome of this multiplication equals the total number of little squares contained within the black-framed rectangle. In the present example, the upper-left black-framed rectangle represents the multiplication 1 × 1, the lower-right one 8 × 8. Now consider, say, the multiplications of 2 × 3, 2 × 4, and 2 × 7. Count the number of little squares inside the black-framed rectangles that correspond to each of these multiplications and note that—as per the distributive law of multiplication—(2 × 3)+(2 × 4) = 2 × 7.

**FIGURE 2:**
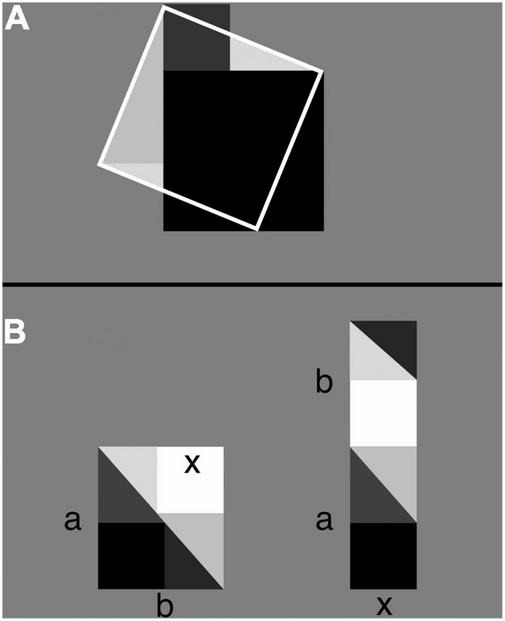
Abstract algebraic expressions as physical geometric puzzles [adapted from Wang (2009)]. **(A)** Let the areas of, respectively, the dark-gray, black, and white-framed squares be *a*^2^, *b*^2^, and *c*^2^. By placing the triangular parts of the dark-colored squares that fall outside the white frame onto the light-colored triangles inside it, the dark-colored squares exactly cover the area of the white-framed square. That is, *a*^2^+*b*^2^ = *c*^2^ (the Pythagorean theorem). **(B)** On the left, a dark square is shown that just fits inside a dark-colored triangle. Task: Find the length *x* of one of the sides of the dark square. Solution: Next to the triangle and square (shown in dark colors) add a copy of it (shown in light colors) so that a rectangle emerges with area *ab*. Rearrange the pieces (shown on the right) so that another rectangle emerges with area (*a*+*b*)*x*. Compare the rectangle on the left with the one on the right and note that *ab* = (*a*+*b*)*x* and thus that *x* = *ab*/(*a*+*b*).

Embodied math education is tapping into this embodied math cognition by providing students with objects and tools that can help them understand abstract mathematical concepts and operations in more concrete physical or virtual ways (Carbonneau et al., 2013; Abrahamson et al., 2020). To give them a better sense of proportion, ratio, fraction, young students have been asked to keep a screen green while moving two cursors up or down (Hutto et al., 2015). The otherwise red screen turned green whenever the students happened to raise their right hand twice as high as their left one. Trial and error then showed the students how, as their hands move up or down, the distance between them had to be proportionally increased or decreased to keep the screen green. A sense of proportion was thus instilled in the students in terms of not abstract symbols and austere rules but concrete physical action and sensory experience (see also Szkudlarek et al., 2022). Hands-on mathematics has a long history and features prominently in Montessori education (Laski et al., 2015). Earlier in the 20th century, Laisant’s table (Laisant, 1915; Maffia and Mariotti, 2020) allowed students to physically explore—by counting squares—the meaning and validity of the distributive law of multiplication (Figure 1). And physically rearranging simple geometrical shapes has been used as far back as during the Han dynasty, 206 B.C.−220 A.D. (Wang, 2009), to prove the Pythagorean theorem and resolve problems like finding the side of an unknown square that just fits a known right triangle (Figure 2; for video demonstrations, see footnote^1^).

One major problem remains, however, a meta-analysis found that whereas hands-on interaction considerably improves retention of abstract mathematical facts or operations, it does not have as big a positive effect on students’ ability to solve new abstract mathematical problems (Carbonneau et al., 2013). Combining embodied and symbolic mathematics helps establish a link between the two and facilitates the desired transfer of learned skills from the one to the other (Laski et al., 2015; Coles and Sinclair, 2019; Maffia and Mariotti, 2020). Still, there obviously remains a gap between embodied cognition and embodied education on the one hand and un-embodied, formal mathematics on the other. The un-embodied, formal mathematics itself is still as opaque and abstract as it has always been.

Like symbolic math, iconic mathematics is rigorous and formally based on axioms but, unlike symbolic math, it is nonetheless much more embodied. That is, it is expressed with the help of either physical or virtual objects, or iconic depictions of objects, rather than with arbitrary tokens that depict nothing. Iconic mathematics may help students transition from embodied to symbolic math by offering them something in between. Yet more importantly, as a coherent system of mathematics in its own right, iconic mathematics also has the potential to become a valid alternative to symbolic mathematics. As we shall see shortly, doing iconic mathematics resembles performing calculations on an abacus. In the West, this age-old tool has all but fallen out of use. In the East, however, it continues to be popular in math education, and as shown in the last section, it has even been found to improve the mathematical performance of students with a severe numerical disability (*dyscalculia*). As a kind of extension of the abacus, iconic math thus offers hope for those who struggle with symbolic math.

## Main Conceptual Analysis: Nuts and Bolts

### Iconic Addition and Subtraction

The mathematics we currently have is more abstract, and less user friendly, than it needs to be. The trouble starts with something even more basic than its axioms: its digits. The problem is that the digits of symbolic math offer no clue to either the meaning of the numbers they represent, the interrelationships between these numbers, or the use of these numbers in calculations. For example, none of the tokens used in “3 + 4” suggest in any way that the end result should be “7.” To be able to see that it should, we need to translate the abstract tokens into something more concrete. In fact, in the absence of anything to hold on to, young children and dyscalculics often try to relate abstract digits to the physical ones of their hands (Geary, 2011; Kucian and von Aster, 2015; Tran et al., 2017). Soon they run out of hands and fingers, and thus out of answers. Engaging additional body parts, New Guinean Yupno men count up to 33—a number represented by the penis and referred to as such (Lancy, 1983; see also Butterworth, 1999; Mareschal et al., 2013). Yupno women, reportedly, do not count in public. It is clear, in any case, that the body-parts system inevitably runs up against its limits.

**FIGURE 3:**
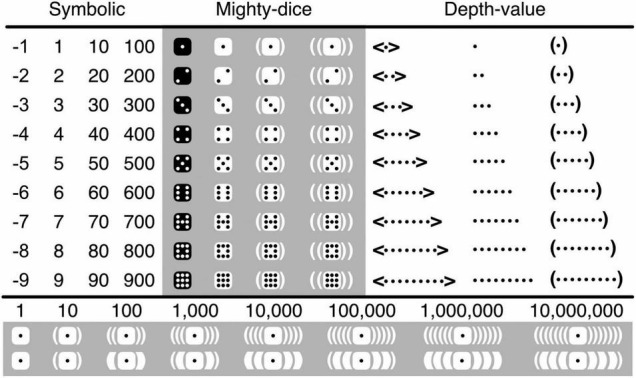
Iconic number representation. Ordinary symbolic numbers are shown along with their iconic translations based on dot tally-marks. Of the mighty-dice translations, all four columns are shown; of the depth-value ones, only the first three. The three rows underneath the main table show symbolic numbers, corresponding iconic numbers, and corresponding iconic numbers that have been adjusted for readability. In the latter, two nested pairs of brackets are replaced by just one pair of thick ones. The might-dice and thin-fat notations, henceforth together referred to as the “black-and-white notation,” have been developed for easy reading; for easy writing of this notation, ideally some kind of an app would be developed.

To represent numbers, a more intuitive alternative to the use of digits, and a more practical one to that of body parts, is the use of tally marks—typically bars or dots (Bricken, 2019a; see also Schwenk et al., 2017). Adopting Kauffman’s (1995) “depth-value notation,” Bricken and James use collections of aligned or unaligned dots (Figure 3). To render them more ergonomic and almost as concise as digits, I organize these dots within the standard configurations of what I call “mighty dice,” which are like ordinary dice but can represent the numbers 1−9 rather than merely 1−6 (Figure 3; Krajcsi et al., 2013). Because symbolic digits are abstract, and their meanings established only by convention, their numeric value (cardinality) must be retrieved from memory. Numbers in the mighty-dice notation, instead, can be read in three different ways. First, one can *retrieve* their cardinality from memory by recognizing the conventional configurations of the dots. Second, one can *subitize* the dots (enumerate them at a single glance with near-perfect accuracy; Anobile et al., 2019; Liu et al., 2020; Decarli et al., 2021), which is facilitated by the dice-like configurations (Krajcsi et al., 2013; Jansen et al., 2014; Katzin et al., 2019). And third, one can *count* the dots, which requires more than a single glance but is nonetheless considerably easier and faster when the dots are configured like either dice (Jansen et al., 2014) or mighty dice (Krajcsi et al., 2013; see also Piazza et al., 2002; Ashkenazi et al., 2013).

Bricken and James represent negative numbers by enclosing them between angle brackets (Figure 3). Yet, black and white are intuitively seen as opposites, and across cultures, perhaps because we are a diurnal rather than nocturnal species, the particularly dark color of black tends to be perceived more negatively than the particularly light color of white (Jonauskaite et al., 2020). Logically, emotional negativity is unrelated to mathematical negativity. Psychologically, however, the former can be exploited as a mnemonic device for the latter. I thus represent positive mighty-dice numbers in black on white, with white as the dominant color, and negative ones in white on black, with black as the dominant color^2^. This representation, which also happens to be more concise than the original one, I call the “black-and-white notation.”

**FIGURE 4:**
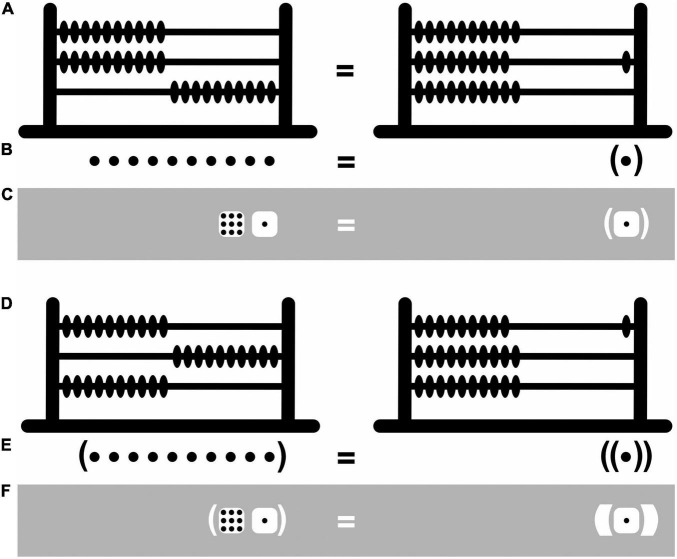
Iconic and abacus number representation. **(A)** Representation of the number 10 with ten beads shifted to the right on the abacus’s lower rung (left) or, equivalently, with one bead with a value of 10 shifted to the right on its next higher rung (right). **(B)** A similar representation in depth-value notation with ten beads (left) or, equivalently, one bead with a value of 10 in a container (right). **(C)** A similar representation in black-and-white notation. **(D)** Representation of the number 100 with ten beads with a value of 10 shifted to the right on the abacus’s second rung (left) or, equivalently, with one bead with a value of 100 shifted to the right on its next higher rung (right). **(E,F)** Similar representations in the depth-value and black-and-white notations.

The depth-value system Bricken and James rely on resembles an abacus—an apparatus that deals with numbers in a particularly concrete and tangible way (Figure 4). The abacus features several rungs of beads, and the higher the rung the larger the numbers it represents. This is fairly intuitive, as high and large go together psychologically in a way that high and small or low and large do not—they have the same *polarity* (Proctor and Cho, 2006). By arbitrary convention still, ten beads on the abacus’s lower rung can be traded in for exactly one bead with a value of 10 on its next higher rung, ten beads with each a value of 10 can be traded in for one with a value of 100 on the next higher rung, and so on. In both the depth-value and mighty-dice systems, the rungs are replaced with “containers” (here pairs of enclosing round brackets). Like big fish tend to eat smaller ones but not the other way around, containers representing larger numbers can encompass containers representing smaller ones but not the other way around. And like in the case of the abacus, ten dots can be traded in for exactly one dot with a value of 10 in a container, ten dots in containers with each a value of 10 can be traded in for one dot with a value of 100 in a container nested within a container, and so on (Figure 4). The numeric value of dots thus increases with the depth of their nesting within containers. The use of the abacus, and of the depth-value and mighty-dice systems, is only partially intuitive and partially still depends on arbitrary conventions. Importantly, however, by appropriately shifting beads from one side to the other on the abacus’s various rungs, addition and subtraction become transparent mechanical processes before our eyes rather than opaque mysterious ones inside our heads. And, by shifting dots in and out of containers, much the same can be achieved in the depth-value and mighty-dice systems.

**FIGURE 5:**
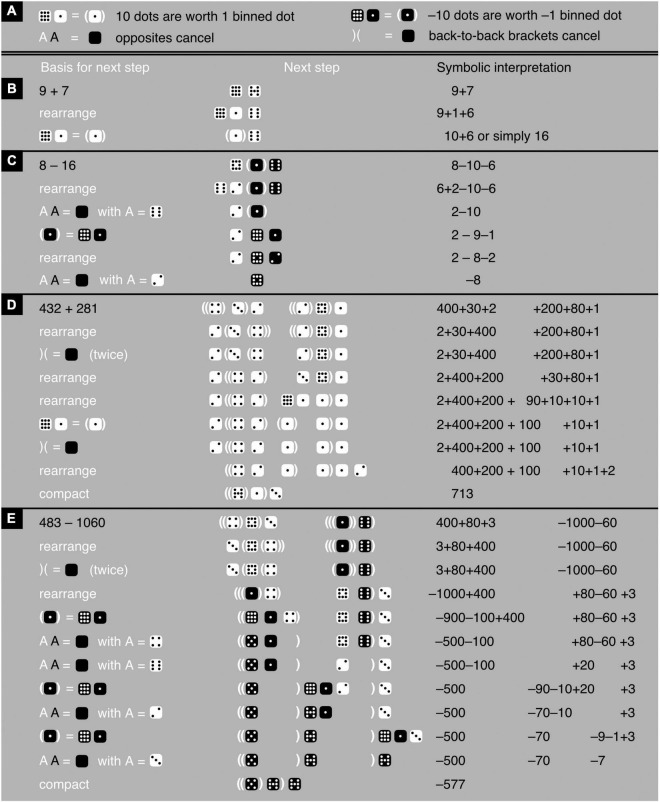
Iconic addition and subtraction. **(A)** Provides the rules, whereby the first rule on the right can be derived from the first two rules on the left. **(B–E)** Shows examples of iconic addition and subtraction with—based on the provided rules—a justification for each next step in a calculation (left), the calculation-step itself (middle), and a symbolic interpretation (right). Note the following: First, the brackets that enclose a number need not be adjacent to this number. For instance, in the 432+281-example, the 4 of 432 is enclosed between two pairs of brackets even though the right-most bracket of one of these two pairs appears immediately to the right of the 3 and not the 4. The 3 itself is enclosed by one pair of brackets. Second, the order of iconic digits (mighty dice) within an iconic number is free and space between these digits optional, like in the final two examples. Third, in the end, just for readability, dots are binned, shifted to the right as much as standard notation allows, and compacted. Fourth, the iconic representation of a number and of an addition can coincide. For instance, in the 9+7-example, the iconic notations of 10+6 and of 16 are identical.

In the latter two systems, more specifically, addition and subtraction consist of putting numbers together and rewriting them to obtain as few numbers as possible (typically just one), each containing as few dots as possible (Figure 5; see also Kauffman, 1995; Bricken, 2019a). Provided standard notation is respected (Figure 3), the dots can be moved, binned (put into containers), and unbinned (taken out of containers), and matching pairs of white and black elements can be eliminated (Figure 5). There is no zero in iconic mathematics; the zero is replaced with literally nothing. When clarity demands some kind of token, in the depth-value notation one can use an empty space between a pair of angle brackets: the negative of nothing is still nothing, just like −0 = 0. In the black-and-white notation, I use an empty black die instead. For anyone unaccustomed to it, dealing with iconic digits and iconic addition and subtraction may at first be a challenge. Yet consider how it compares to learning symbolic digits and symbolic addition and subtraction for the first time.

### Iconic Arithmetic and Algebra

The abacus can handle not only addition and subtraction but also multiplication and division. I will not go into details here but instead lay out a system that reminds one again of the abacus, with its rungs replaced by containers. Unlike the abacus, this system can not only handle arithmetic (with numbers) but also algebra (with at least one variable instead of a number). This *James algebra* (James, 1993; Bricken, 2019a,b, 2021) features negation, addition, subtraction, exponentiation, and taking logarithms but has no need for explicit multiplication or division.

Multiplication of natural numbers is effectively a shorthand for repeated addition. Starting from 0, for example, adding 10 three times (10 × 3 = 30), or 3 ten times (3 × 10 = 30), gives us 0+10+10+10 = 30 or 0+3+3+3+3+3+3+3+3+3+3 = 30. The inverse of multiplication is division, and division of natural numbers is effectively a shorthand for repeated subtraction^3^. Starting from 30, subtracting 10 three times (30/10 = 3), or 3 ten times (30/3 = 10), gets us back to 30−10−10−10 = 0 or 30−3−3−3−3−3−3−3−3−3−3 = 0. Exponentiation can function as a shorthand for repeated multiplication—a multiplication by itself of a number (the “base”), repeated as many times as indicated by a another number (its “power”). Starting from 1, for example, multiplying by 10 three times gives us 1 × 10 × 10 × 10 = 10^3^ = 1000, with 10 representing the base of 10^3^ and the superscript 3 its power. The inverse of raising a base to a certain power is taking the logarithm of its outcome: log_10_(1000) = log_10_(10^3^) = 3, with 3 representing the logarithm in log_10_(1000) = 3 and the subscript 10 its base. If the power is not larger than 1 but between 0 and 1, exponentiation can function as a shorthand for repeated division. Starting from 1000, dividing by 10 (1000^1/3^ = 10) three times (log_10_(1000) = 3) gets us back to ((1000/10)/10)/10 = 1. This kind of exponentiation is also called to taking roots and $1000^{1/3}=\sqrt[{3}]{1000}=10$.

Note that 1 × 10 × 10 × 10 = 1000 can be written as 10^0^× 10^1^× 10^1^× 10^1^ = 10^3^ and also, without using any multiplication, as 10^0+1+1+1^ = 10^3^. Likewise, ((1000/10)/10)/10 = 1 can be written as ((10^3^/10^1^)/10^1^)/10^1^ = 10^0^ and also, without using any division, as 10^3–1–1–1^ = 10^0^. Log_10_ is often abbreviated to log, and so, log(10) = 1, log(100) = 2, and log(10 × 100) = log(1000) = 3. What this means is that 100 × 10 can be rewritten as 10^log(100 × 10)^, which equals 10^log(1000)^ and thus 10^3^. Yet, importantly, using addition rather than multiplication, 100 × 10 can also be rewritten as 10^log(100)+ log(10)^, which equals 10^2+1^ and thus also 10^3^. Likewise, 100/10 can be rewritten as 10^log(100/10)^, which equals 10^1^. Yet, importantly, using subtraction rather than division, it can also be rewritten as 10^log(100)–log(10)^, which equals 10^2–1^ and thus also 10^1^. In fact, powers and logarithms can transform any multiplication or division into an addition or subtraction. So, to avoid bringing more operators into play than necessary, James algebra uses the iconic equivalents of powers and logarithms but no equivalents of multiplication or division.

**FIGURE 6:**
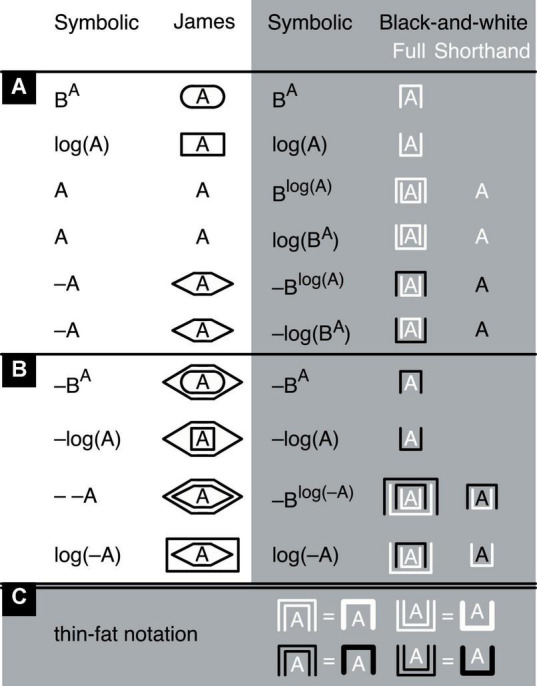
Definitions. In black on white (left), symbolic operations are shown with their James-algebraic translations. In black or white on gray (right), equivalent symbolic operations are shown with their full black-and-white translations and shorthand versions. These shorthands are merely a convenience and can at any time be replaced by their full versions. **(A)** Basic operations. **(B)** Some operations derived from the basic ones. **(C)** Equivalent fat notations of the exponent of an exponent of A and the logarithm of a logarithm of A (white), as well as their negative versions (black). The logarithms and exponents are assumed to have the same arbitrary base B. Lacking a better alternative, variables are still represented by letters.

In symbolic mathematical notation, the interrelationships between mathematical operations are not very apparent and must be learned and stored in memory. Instead of a letter string like “log” for logarithm or seemingly unrelated tiny superscript to express the power of a number, James algebra expresses the power or logarithm of a number by putting it into one or another container. This is a rather arbitrary choice, but containers do have the advantage of being concrete and easy to picture rather than abstract and further removed from sensory experience (Figure 6, right; Bricken, 2019a). Indeed, one could in principle use physical containers (and physical mighty dice) instead of their depictions; this might be an especially good idea to let very young children get the hang of their use (Hutto et al., 2015; Tran et al., 2017). Bricken presents various alternatives to containers, including blocks, nodes in networks, and even entire rooms. These can all be turned into physical objects, concrete electronic devices, or immersive virtual-reality worlds. For writing convenience, Bricken most often simply uses pairs of brackets that are merely suggestive of containers. A pair of square brackets, for example, serve as a logarithm operator, a pair of round ones as a power operator (Supplementary Figure 1). Note, however, that round brackets also appear, with a different meaning, in the depth-value notation of numbers (Figure 3). To allow an unambiguous use of the depth-value notation within James algebra, and also to reduce container nesting and enhance readability, I therefore propose an alternative notation that extends the previous section’s black-and-white one.

The extended black-and-white system relies on two independent binary operators (Figure 6; for an alternative notation, see Supplementary Figure 1). One is a container operator that either takes the logarithm of a number (by putting this number into an upright container) or raises it to a power (by putting it into an upside-down container). The other is a contrast operator that gives a number a value that is either positive (light) or negative (dark). If color can be used, upright containers could, instead of black or white, be yellow or blue (easily distinguished by almost all colorblind people). This would enhance the perceptual difference between upright and upside-down containers and thereby improve readability (see Supplementary Figures 3, 4). Alternatively, hue could also be an interesting option for exploitation as a third operator, should one be desired. In this case, the same container could concurrently express three different operators with its orientation (upright vs. upside down), contrast (light vs. dark), and hue (yellow vs. blue, whereby brown counts as dark yellow).

I will assume that numbers only exist in combination with their operators, just like −1, as a negative number, cannot exist without the negation operator and ½, as a rational number, not without the division operator. Because the container operator is assumed to be binary (as simple as possible and easy to implement in electronic devices), it can only take a logarithm or raise a number to a power, it cannot in addition leave a number unmodified. Yet taking the logarithm of a number (by putting the number into an upright container), and raising the result to a power (by putting the upright container with its content into an upside-down container), gives us something equivalent to A. For convenience and for short, I will label this A-equivalent simply “A” (Figure 6, “shorthand”-column). Conversely, first raising a number to a power and then taking the logarithm of the result also gives us something equivalent to A, which I will therefore also label “A.”

**FIGURE 7:**
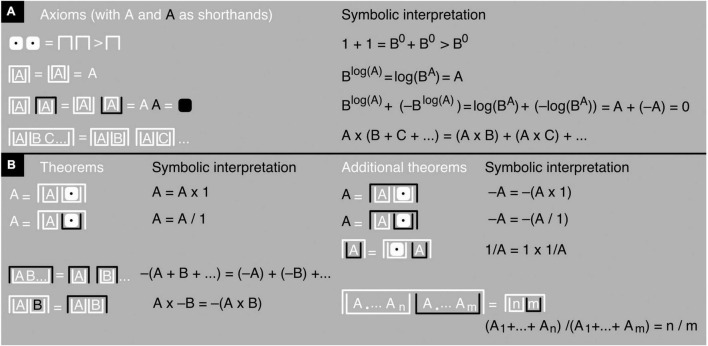
Iconic math’s axioms and a few iconic theorems. **(A)** The axioms of James algebra (slightly altered) shown in black-and-white notation (left) and accompanied by a symbolic interpretation (right). The first line says that 1+1>1, which means 1+1 adds up to more than 1 (namely, 2). It follows that 1+1+1 adds up to even more (namely, 3) and so on. **(B)** Iconic theorems and additional ones added by me myself, derived from the iconic axioms shown in **(A)** and accompanied by symbolic interpretations. In the last additional iconic theorem, an A with a dot as a subscript should be read as A_1_, meaning the first copy of A, and A_*n*_ and A_*m*_ as the nth and mth copies of A. Intervening dots stand for intervening copies of A. So, the theorem shows *n* copies of A in the first upright container and *m* copies of A in the second one.

In this line of thought, the mighty-dice numbers should all be considered shorthands. In fact, it is possible to even *define* the number 1 as an empty upside-down white container (Figure 7; see also Bricken, 2019a); its equivalent in symbolic terms is B^0^, which—regardless of the value of B—equals 1. Similarly, considering that the logarithm of smaller-and-smaller positive numbers approaches negative infinity, one can define −∞ as a completely empty white upright container and ∞ as a completely empty black upright container (see also Bricken, 2019a).

**FIGURE 8:**
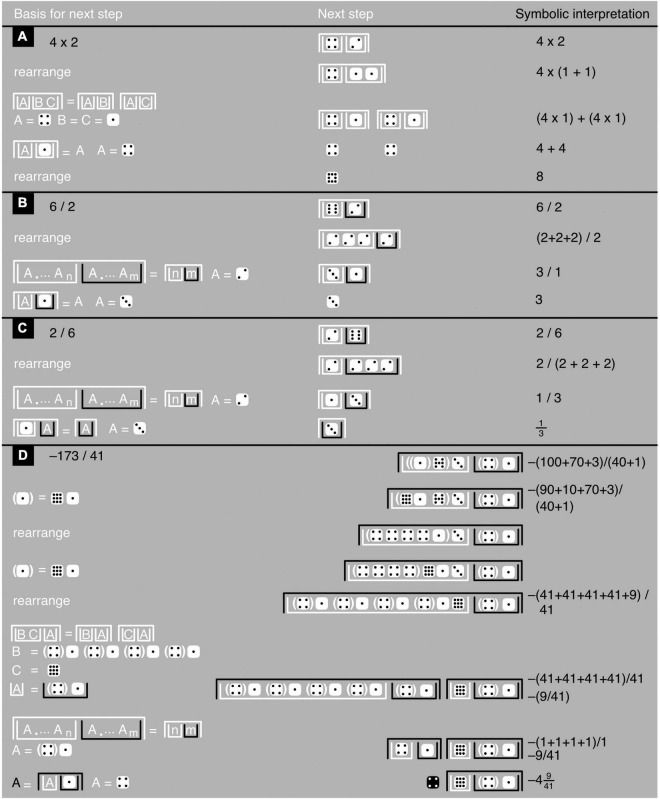
Iconic multiplication and division. **(A–D)** Show examples with, based on Figure 7 axioms and theorems, the justification of each next step toward a problem’s solution (left), the step itself (middle), and a symbolic interpretation (to avoid excessive clutter, the last example has only a few, key symbolic interpretations). The last example shows how to deal with relatively large numbers and with a division’s irreducible remainder. All logarithms and exponents are assumed to have the same arbitrary base.

**FIGURE 9:**
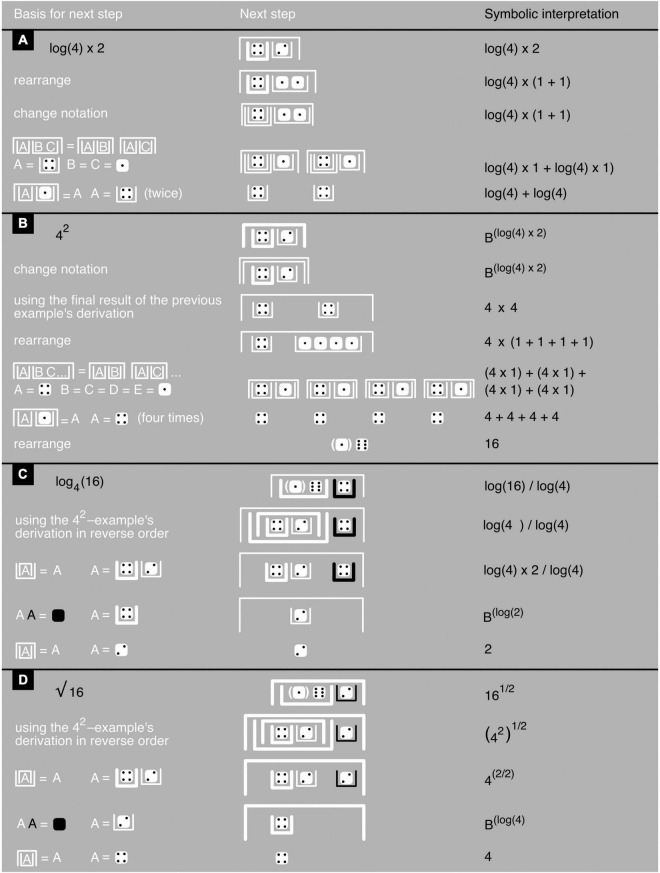
Iconic exponentiation and taking iconic logarithms and roots. **(A–D)** Show examples with, based on Figure 7 axioms and theorems, the justification of each next step toward a problem’s solution (left), the step itself (middle), and a symbolic interpretation (right). In the first example, the logarithm’s base has been left unspecified and the final result cannot be computed. In the third, a base has been specified in the symbolic version, and a *base-free* equivalent of it in the iconic version; now the final result can be computed.

**FIGURE 10:**
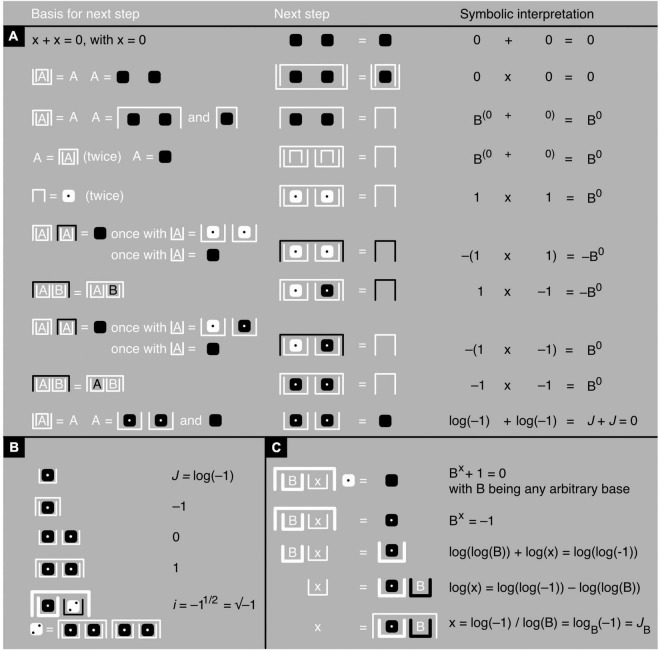
Imaginary number *J*. **(A)** An iconic proof of the fact that *x* = *J* is a solution of the equation *x* + *x* = 0, with, based on Figure 7 axioms and theorems, the justification of each next step toward a problem’s solution (left), the step itself (middle), and a symbolic interpretation (right). **(B)** An iconic definition of *J* and iconic definitions, exclusively in terms of *J*, of the other candidate foundational numbers: –1, 0, 1, and *i*. **(C)** An iconic proof of the fact that x = *J*_*B*_ solves the equation B*^x^* + 1 = 0, with B being any arbitrary base, which for example can be *e*. Note that mixing real and imaginary numbers is subject to restrictions (Bricken, 2021), and to prevent inconsistencies, the last axiom in Figure 7 is not used here.

The contrast operator only affects the outer container of a number or mathematical expression, not the container’s contents. This procedure avoids confounding numbers and expressions that need to be distinguished, like for example −A and – –A (Figure 6). Note that, in the black-and-white notation, iconic A and iconic –A differ only in contrast, not in container type; and iconic B*^A^* and iconic log(A) only in container type, not in contrast. This demonstrates the independence of the contrast and container operators. In principle, instead of two, just one operator would suffice—an operator that “marks” a single, fundamental distinction between yin and yang, so to speak, between something and nothing, contained and uncontained, black and white, and—in electronic devices—on and off (Spencer-Brown, 1969; see also Kauffman and Varela, 1980; Kauffman, 1995; Bricken, 2019a,b, 2021). However, reducing operator types tends to come at the price of more container nesting or otherwise reduced readability (Bricken, 2019b, Chapter 20.5). For this reason, I will stick to a two-operator system here (see also Kauffman, 1995; Bricken, 2019b). All permissible transformations of iconic mathematical expressions, as well as the meaning of addition, are spelled out in the axioms and theorems provided in Figure 7 (for a proof of the last additional theorem, see Supplementary Figure 2). The application of these axioms and theorems is illustrated with basic arithmetic examples in Figures 8–10 and with an algebraic example in Figure 10C (for another example, see Supplementary Figure 2).

Although James algebra does not use multiplication or division in any explicit way, its iconic power-and-logarithm equivalents of these operations are nonetheless easily interpreted in terms of multiplication and division. For example, two or more white upright containers nested within one white upside-down container represent a multiplication (see the 4 × 2-example in Figure 8). These white upright containers can also be seen as the numerator of a division in which any black upright containers take the role of denominators (see the rest of Figure 8). Iconic formulations, unlike symbolic ones, are thus often easy to interpret in multiple, mathematically equivalent ways, bringing more clearly to the fore the interrelationships between these different interpretations (Figures 8–10).

Powers and logarithms in James algebra differ a little from those most used in symbolic mathematics. In B*^x^* and log_B_(x), B is the base of these expressions. It so happens, however, that expressions like these can always be rewritten in a form in which the base can have any arbitrary value rather than just a single specific one. For example, log_4_(16) = log_B_(16)/log_B_(4) regardless of the value of B. To keep things simple, James algebra therefore only uses “base-free” expressions in which, for example, log_4_(16) is replaced—without ever mentioning any base—with an iconic equivalent of log_B_(16)/log_B_(4) that does not use explicit division (Figure 9C; see also Figure 10C).

All beginnings are a challenge, and already familiar symbolic mathematics will, of course, be easier than as yet unfamiliar iconic mathematics. To compare them fairly, therefore, put yourself in the shoes of someone to whom both are new. Imagine you know nothing and have to start from scratch.

### Iconic Imaginary Numbers and Trigonometry

James algebra can deal not only with mundane topics but also with exotic ones that might seem more challenging to bring down to earth. For example, due to their particular graphical nature, iconic mathematical expressions can sometimes look a bit like modernistic paintings. It is symbolic mathematics, however, that produced a gravity-defying equation that many consider conceptual art: *e*^*i*π^ + 1 = 0 (*Euler’s identity*). The formula relates to one another no fewer than five mysterious numbers. The first two are 0 and 1, which have rather unusual properties (Bricken, 2019b,^2021^). The next two are *e* and π, two numbers that, by definition, are impossible to express as rational ones, and are thus considered *irrational*, and that cannot even be described with elaborate algebraic operations and are thus also considered *transcendental*. The letter *e* stands for “Euler’s number” (the base of the “natural” logarithm, discovered by Bernoulli rather than Euler) and the Greek letter π for the ratio of a circle’s perimeter to its diameter. The fifth number is *i*, an *imaginary* number defined as *i* = √–1. Because *i*^2^ = −1, the number *i* is an imaginary solution to such equations as *x*^2^ + 1 = 0.

A less well-known imaginary number that is also associated with Euler’s identity is *J*, defined as *J* = log(–1). Defying common sense, *J* turns out to be a nonzero solution of the equation *x* + *x* = 0. Substituting x with *J*, the equation becomes *J* + *J* = log(–1) + log(–1) = log(–1 × –1) = log(1) = 0 (for an iconic proof, see Figure 10; see also Bricken, 2021). Thus, although *J* is nonzero and therefore certainly counts for something, two *Js* together add up to nothing (in iconic math) or zero (in symbolic math). Adding two *J*s together is like going around a circle 180° (something) and then another 180° (something) to get back to 0° (nothing) or like taking a step toward a mirror and then an imaginary step into the mirror, which reflects you back to where you came from—square zero (Bricken, 2021). Although two *J*s add up to nothing, *J* can nonetheless function as the very cornerstone of arithmetic, as all other numbers can be derived from it, in particular those that are candidate foundational numbers themselves (Figure 10B; Bricken, 2021). Importantly, just like log_2_(–1) solves the equation 2*^x^* + 1 = 0 and log_3_(–1) the equation 3*^x^* + 1 = 0, log_*e*_(–1) solves the equation *e*^*x*^ + 1 = 0. This means that *e*^*i*π^ + 1 = 0 (Euler’s identity) is a special case of B*^x^* + 1 = 0, popping up when B = *e* and x = *J*_*e*_ = log_*e*_(–1) = *i*π (for iconic representations of *i*, *J*, and *J*_*B*_ see Figure 10; for iconic representations of *i*, π, *e*, and Euler’s identity, see Bricken, 2021).

Euler’s identity is better known as a special case of not B*^x^* + 1 = 0 but *Euler’s equation*: *e*^*i*α^ = cos(α) + *i* sin(α), emerging when α = π. Euler’s equation implies that all three of the fundamental functions of trigonometry can be expressed as exponential ones instead: cos(α) = (*e*^*i*α^ + *e*^–iα^)/2, sin(α) = (*e*^*i*α^ − *e*^–iα^)/2*i*, tan(α) = sin(α)/cos(α). This, in turn, means that James algebra needs no other tools than those already discussed to be able to deal with trigonometry (Bricken, 2021). Very little extra is needed, in fact, to allow it to handle differential calculus as well (Bricken, 2021). This goes to show that there is no reason to dismiss out of hand the idea that, in principle, all of mathematics can be iconized.

## Beneficiaries: Hypercalculics Versus Dyscalculics

Now that the background and nuts and bolts of iconic mathematics have been laid out, the question arises who stands to benefit from this alternative, more concrete, more grounded system of mathematics. Who needs it and why? I lay out how a comparison between the broader psychological profiles of hypercalculics and dyscalculics suggests that the people traditional mathematics leaves behind tend to have a problem that, compared to symbolic mathematics, iconic mathematics is better equipped to handle.

### The Problem

Intriguingly, whereas some intellectually disabled “savants” can effortlessly perform extraordinary calculations off the top of their heads (Treffert and Christensen, 2005; Baron-Cohen et al., 2007; Bor et al., 2007; Crespi and Badcock, 2008; Badcock, 2009, 2019; Heavey et al., 2012; van Leeuwen et al., 2020), quite a few properly schooled and otherwise intelligent people can hardly manage any math at all. About 3–6% of otherwise normal children, for example, are afflicted with dyscalculia and have unusually poor numerical skills (Dehaene, 2011; Kucian and von Aster, 2015; Butterworth, 2019; see also Kaufmann et al., 2013). Dyscalculics find it difficult to estimate quantities, understand what numbers mean, and perform basic calculations (Kucian and von Aster, 2015). Children with mathematical learning disabilities, who may have dyscalculia, run into trouble in telling ways (Geary, 2011). To solve the equation 5 + 3 = ?, for example, one can count five times “1, 2, 3, 4, and 5” and then three more times: “6, 7, and 8.” This method tends to be preferred by the least talented children. A quicker method lets one start from 5 right away and then count only “6, 7, and 8.” Unlike most of their peers, however, some children undercount with “5, 6, and 7.” Others, trying to subtract a larger number from a smaller one, arrive at 83−44 = 41, subtracting 4 from 8 but 3 from 4 rather than the converse, or they misunderstand “borrowing” and arrive at 92−14 = 88 rather than 78.

### The Proximate Cause

The problems of dyscalculics appear related to temporary or permanent weaknesses in one or more cognitive or perceptual domains (and deficits in associated brain regions; Dehaene, 2011; Geary, 2011; Kaufmann et al., 2013; Kucian and von Aster, 2015; Rapin, 2016; Menon and Chang, 2021). The problems concern *visual-spatial ability*, hence numerical representation; *working memory and attention*, hence mathematical reasoning and the maintenance and manipulation of quantities (see also Friso-Van den Bos et al., 2013); *semantic memory*, hence the storage of mathematical facts; and *procedural memory*, hence the acquisition of mathematical skills, as opposed to knowledge (see also Ullman et al., 2020). Some doubt has even been cast on ordinary people’s mnemonic abilities. At remembering briefly presented symbolic numbers, in fact, most of us are far worse than a well-trained chimpanzee (Matsuzawa, 2009; to take the test, search “chimpanzee memory” on YouTube). Of note, in any case, is that there is substantial comorbidity between dyscalculia and other disorders, in particular dyslexia—delayed and deficient reading despite an otherwise normal cognitive ability (Geary, 2011; Butterworth and Kovas, 2013; Kucian and von Aster, 2015; Ullman et al., 2020).

The talents of hypercalculics are accompanied by a mirror opposite psychological profile to that of dyscalculics. Kim Peek, for example, who inspired the movie “Rain man,” was able to tell within seconds the day of the week on which people were born (superior calculation skill) and remember the contents of more than 9,000 books (superior memory), which he read at a speed of about 9 s per page (hyperlexia: high-speed and precocious reading) (Treffert and Christensen, 2005; Badcock, 2009, 2019; see also Heavey et al., 2012). In contrast, he had poor communication skills, was socially inept, and could not live without his father’s constant help (Treffert and Christensen, 2005; Badcock, 2009, 2019).

Much better adjusted to society, but like most hypercalculics diagnosed with autism (although only in its mild form of Asperger’s) is the savant Daniel Tammet (Baron-Cohen et al., 2007; Bor et al., 2007; Badcock, 2009, 2019; van Leeuwen et al., 2020; see also the BBC documentary “The boy with the incredible brain”). Tammet can learn a new language in a week, perform complex mental calculations in seconds, and recite 22,514 decimals of the number π. To Tammet, abstract numbers are not really abstract; they evoke in him percepts of concrete shapes—a form of synesthesia (sensory experience unprovoked by commensurate sensory input), which is a condition associated with autism (Baron-Cohen et al., 2007; van Leeuwen et al., 2020, 2021). According to Tammet, it is the phantom number-shapes he sees that help him pull off his startling numerical feats. Whether other hypercalculics also visualize abstract numbers in concrete ways is currently unknown but several studies confirm that synesthesia does influence the processing of not only concrete numerosities but also abstract numbers (for a brief review, see Gertner et al., 2013).

### The Ultimate Cause

According to the diametric theory of genomic imprinting, people’s mental strengths and weaknesses are shaped by a tug of war between their parents (see Bressan and Kramer, 2021 and references therein, including especially Crespi and Badcock, 2008; Badcock, 2009, 2019; Del Giudice et al., 2010; Crespi, 2020; see also Úbeda, 2008; Úbeda and Gardner, 2015; Mokkonen et al., 2018). As parents bestow their genes onto their children, some of these genes are turned on and others off. Remarkably, in the case of so-called imprinted genes, the maternally and paternally inherited copies show a diametrically opposite pattern of activation and silencing (Moore and Haig, 1991; Haig, 2010; Kotler and Haig, 2018). Genomic imprinting likely evolved for the benefit of the individual parent rather than their offspring (and possibly also to turn off viral DNA that is permanently embedded within the offspring’s own DNA; Kramer and Bressan, 2015). As it normally leaves the genes of only one parent expressed, imprinting locally annuls the offspring’s benefit of inheriting genes from two parents rather than just one. Much the same is true for sex chromosomes, especially in men (Xirocostas et al., 2020). Compared to others, imprinted genes and sex chromosomes are indeed disproportionately often implicated in both physical and mental disease, most prominently in pairs of syndromes that are genetically related but have roughly opposite physical and behavioral characteristics (Bressan and Kramer, 2021).

Some imprinted genes promote the offspring’s consumption of maternal resources during gestation (Moore and Haig, 1991; Haig, 2010; Kotler and Haig, 2018) and the growth of parts of the brain that allow one to deal with the physical environment (Bressan and Kramer, 2021). The paternally inherited copies of these genes tend to be turned on and the maternally inherited ones off. Some imprinted genes promote the growth of parts of the brain that allow the offspring to deal with its social environment, which also facilitates its ability to take the mother’s directions. The maternally inherited copies of these genes tend to be turned on and the paternally inherited ones off. A relatively strong semantic memory for facts (including technical ones) but weak episodic memory for events (including social ones) tends to emerge whenever paternal imprinting dominates; the opposite whenever maternal imprinting does (Bressan and Kramer, 2021). A paternal imprinting bias is associated with a tendency toward autism-spectrum disorders, which besides autism includes *hyperlexia* (Ostrolenk et al., 2017), despite that other verbal skills tend to be poor. A maternal bias, instead, is associated with a tendency toward psychosis-spectrum disorders, which besides schizophrenia includes *dyslexia*, despite that other verbal skills tend to be good. Dyslexia is frequently comorbid with dyscalculia and savantism with hypercalculia. This circumstance leads one to suspect that dyscalculia may be a psychosis-spectrum feature and hypercalculia an autism-spectrum one. In fact, because of its strong association with autism, savantism is frequently called “autistic savantism.”

Even disregarding full-blown mental disorders, individuals with just a slight tendency toward autism are more likely to be interested in math, and to be better at it, than individuals with a slight tendency toward psychosis (Bressan and Kramer, 2021). Among people with an autistic tendency, there are relatively many men (Bressan, 2018) and, among those with a psychotic tendency, relatively many women (Bressan and Kramer, 2021). Overall, men do not outperform women in math, but among the best performing are relatively many men (Wang and Degol, 2017). Moreover, better math than verbal performance predicts greater affinity with, and success in, math and the physical sciences (Wang and Degol, 2017; see also Su et al., 2009). Parental and societal expectations and pressures seem important (Wang and Degol, 2017) but the reason behind their existence is still unclear. In fact, countries with the greatest gender equality in such things as income, parliamentary seats, and academic enrollment (Norway, Sweden, and Finland) do not have the highest, but the lowest, percentage of women with college degrees in mathematics and the physical sciences compared to other disciplines (Stoet and Geary, 2018, 2020). What the dyscalculia and hypercalculia research and the diametric theory together suggest, in any case, is that math ought to be easier for the least talented among us if it were less of a burden on reading ability and (non-episodic) memory.

### The Solution

Requiring no reading and hardly any memorization, the abacus accommodates these needs perfectly. No wonder it has been in continuous use for more than 4,000 years (Ifrah, 2001). Interestingly, learning to perform mental calculations with an imaginary, rather than a real, abacus has been associated with functional and structural changes in visuospatial and frontoparietal areas of the brain, with related improvements in working and short-term memory, numerical magnitude processing, and calculation performance (review: Wang, 2020; recent papers: Lu et al., 2021; Zhang et al., 2021) as well as with a reduction in dyscalculia (Lu et al., 2020). Several studies found a greater practice effect of abacus-based mental calculation than of additional course work in symbolic mathematics (Wang, 2020). When they compare which of two abacus depictions has more beads, children trained in abacus-based mental calculation are distracted by the beads’ positions on the abacuses’ rungs (Du et al., 2014). These positions are task-irrelevant but do affect the beads’ numerical value. The finding thus demonstrates that bead arrangements can fully automatically invoke associations with cardinality.

The abacus is ill-suited to dealing with powers and logarithms and cannot handle algebra. As a sophisticated kind of abacus, iconic mathematics is much more versatile; yet it can be seen as a natural extension of the abacus and it still mimics the features that brought the abacus success.

## Discussion: Main Benefits of Iconic Mathematics

To better understand mathematics, it can help to change one’s perspective of it. The solution of many an algebraic problem, for example, seems more apparent when this problem is rephrased as an equivalent geometric one. Likewise, that nonzero numbers can add up to zero is difficult to wrap one’s head around, until one thinks of them as steps along a circle that bring you back to where you came from. Iconic mathematics offers a different perspective on math than symbolic mathematics does, and this can be instructive for the same reason. Changing perspective also helps keep one’s mathematical thinking flexible.

It is often said that mathematics needs to be understood rather than learned by heart. The more exotic the math, however, the less intuitive its axioms, and more generally, theorems only make sense if one manages to recall their derivations, which can be a tall order. In fact, as laid out in the previous section, remembering abstract mathematical facts that on the surface may seem senseless is a major challenge to those who underperform in math. Iconic mathematics can help out by making mathematical expressions more concrete, more intuitive, easier to picture, or even more tangible, and thus—for all these reasons—more memorable.

Like a game of chess or checkers, math is a rule-based game. The rules of chess and checkers are quite arbitrary. To be able to play, however, one need not make sense of these rules, one merely needs to accept them, get used to them, make them second nature. Some games are, of course, easier to learn than others, and the game of mathematics is quite complex and requires extensive training. Whether in a game-like simulator or for real, flying an airplane is complex and requires extensive training too. Yet it is understood that what suffices to avoid accidents are neither technically perfect airplanes nor optimal pilot training; to keep them in the air, airplanes need to be designed to respect their pilots’ perceptual, attentional, and cognitive limitations. Human factors research has, in fact, greatly improved the design of airplanes and countless other products. Yet, although the faulty use of mathematics can certainly have disastrous consequences, math’s ergonomic design is hardly ever questioned. We put all our eggs in the basket of education and hope for the best. No wonder that even for many intelligent and properly schooled individuals, math is not a pair of wings but merely a plane crash waiting to happen. Iconic mathematics, instead, offers hope that we may be able to mold not only users’ brains to the requirements of mathematics, but also mathematics to the requirements of users’ brains.

## Author Contributions

The author confirms being the sole contributor of this work and has approved it for publication.

## Conflict of Interest

The author declares that the research was conducted in the absence of any commercial or financial relationships that could be construed as a potential conflict of interest.

## Publisher’s Note

All claims expressed in this article are solely those of the authors and do not necessarily represent those of their affiliated organizations, or those of the publisher, the editors and the reviewers. Any product that may be evaluated in this article, or claim that may be made by its manufacturer, is not guaranteed or endorsed by the publisher.

## Footnotes

1
https://en.wikipedia.org/wiki/Pythagorean_theorem

2 Perceptually, the surface of the mighty-dice extends behind their dots (a process called *amodal completion*); the color of this surface thus dominates that of the dots, even in the case of the numbers 9 and −9.

3 Multiplication of fractions (numbers divided by other numbers) represents a mix of repeated addition and subtraction. For example: $\frac{2}{3}\times \frac{9}{3}=\frac{2\times 9}{3\times 3}=\frac{9+9}{3+3+3}=\frac{18}{9}=2$ because, to get to zero, one can subtract 9 from 18 twice.

## References

[B1] Abrahamson D. Nathan M. J. Williams-Pierce C. Walkington C. Ottmar E. R. Soto H. (2020). The future of embodied design for mathematics teaching and learning. *Front. Educ.* 5:147. 10.3389/feduc.2020.00147

[B2] Anobile G. Arrighi R. Burr D. C. (2019). Simultaneous and sequential subitizing are separate systems, and neither predicts math abilities. *J. Exp. Child Psychol.* 178 86–103. 10.1016/j.jecp.2018.09.017 30380457

[B3] Ashkenazi S. Henik A. (2010). A disassociation between physical and mental number bisection in developmental dyscalculia. *Neuropsychologia* 48 2861–2868. 10.1016/j.neuropsychologia.2010.05.028 20594996

[B4] Ashkenazi S. Mark-Zigdon N. Henik A. (2013). Do subitizing deficits in developmental dyscalculia involve pattern recognition weakness. *Dev. Sci.* 16 35–46. 10.1111/j.1467-7687.2012.01190.x 23278925

[B5] Badcock C. (2009). *The Imprinted Brain: How Genes Set the Balance Between Autism and Psychosis.* London: Jessica Kingsley Publishers. 10.2217/epi.11.19 22122342

[B6] Badcock C. (2019). *The Diametric Mind: New Insights Into AI, IQ, The Self, and Society.* Tallinn: TLU Press.

[B7] Baron-Cohen S. Bor D. Billington J. Asher J. Wheelwright S. Ashwin C. (2007). Savant memory in a man with colour form-number synaesthesia and Asperger syndrome. *J. Conscious. Stud.* 14 237–251.

[B8] Barrocas R. Roesch S. Dresen V. Moeller K. Pixner S. (2020). Embodied numerical representations and their association with multi-digit arithmetic performance. *Cogn. Process.* 21 95–103. 10.1007/s10339-019-00940-z 31701377

[B9] Bor D. Billington J. Baron-Cohen S. (2007). Savant memory for digits in a case of synaesthesia and Asperger syndrome is related to hyperactivity in the lateral prefrontal cortex. *Neurocase* 13 311–319. 10.1080/13554790701844945 18781431

[B10] Bressan P. (2018). Systemisers *are* better at maths. *Sci. Rep.* 8:11636. 10.1038/s41598-018-30013-8 30072765 PMC6072767

[B11] Bressan P. Kramer P. (2021). Mental health, mitochondria, and the battle of the sexes. *Biomedicines* 9:116. 10.3390/biomedicines9020116 33530498 PMC7911591

[B12] Bricken W. M. (2019a). *Iconic Arithmetic Volume I: The Design of Mathematics for Human Understanding.* Snohomish, WA: Unary Press.

[B13] Bricken W. M. (2019b). *Iconic Arithmetic Volume II: Symbolic and Postsymbolic Formal Foundations.* Snohomish, WA: Unary Press.

[B14] Bricken W. M. (2021). *Iconic Arithmetic Volume III: The Structure of Imaginary and Infinite Forms.* Snohomish, WA: Unary Press.

[B15] Butterworth B. (1999). *The Mathematical Brain.* London: Macmillan.

[B16] Butterworth B. (2019). *Dyscalculia – From Science to Education.* New York, NY: Routledge.

[B17] Butterworth B. Kovas Y. (2013). Understanding neurocognitive developmental disorders can improve education for all. *Science* 340 300–305. 10.1126/science.1231022 23599478

[B18] Carbonneau K. J. Marley S. C. Selig J. P. (2013). A meta-analysis of the efficacy of teaching mathematics with concrete manipulatives. *J. Educ. Psychol.* 105 380–400. 10.1037/a0031084

[B19] Coles A. Sinclair N. (2019). Re-thinking ‘concrete to abstract’ in mathematics education: towards the use of symbolically structured environments. *Can. J. Sci. Math. Technol. Educ.* 19 465–480. 10.1007/s42330-019-00068-4

[B20] Crespi B. Badcock C. (2008). Psychosis and autism as diametrical disorders of the social brain. *Behav. Brain Sci.* 31 241–261. 10.1017/S0140525X08004214 18578904

[B21] Crespi B. J. (2020). Why and how imprinted genes drive fetal programming. *Front. Endocrinol.* 10:940. 10.3389/fendo.2019.00940 32117048 PMC7025584

[B22] Decarli G. Paris E. Tencati C. Nardelli C. Vescovi M. Surian L. (2021). Impaired large numerosity estimation and intact subitizing in developmental dyscalculia. *PLoS One* 15:e0244578. 10.1371/journal.pone.0244578 33382740 PMC7774972

[B23] Dehaene S. (2011). *The Number Sense: How the Mind Creates Mathematics.* New York, NY: Oxford University Press.

[B24] Del Giudice M. Angeleri R. Brizio A. Elena M. R. (2010). The evolution of autistic-like and schizotypal traits: a sexual selection hypothesis. *Front. Psychol.* 1:41. 10.3389/fpsyg.2010.00041 21833210 PMC3153759

[B25] Du F. Yao Y. Zhang Q. Chen F. (2014). Long-term abacus training induces automatic processing of abacus numbers in children. *Perception* 43 694–704. 10.1068/p7625 25223112

[B26] Fischer M. H. Brugger P. (2011). When digits help digits: spatial–numerical associations point to finger counting as prime example of embodied cognition. *Front. Psychol.* 18:177–183. 10.3389/fpsyg.2011.00260 22028696 PMC3198540

[B27] Fischer M. H. Glenberg A. M. Moeller K. Shaki S. (2021). Grounding (fairly) complex numerical knowledge: an educational example. *Psychol. Res.* 10.1007/s00426-021-01577-4 34757438

[B28] Fischer M. H. Shaki S. (2018). Number concepts: abstract and embodied. *Philos. Trans. R. Soc. B Biol. Sci.* 373:20170125. 10.1098/rstb.2017.0125 29914993 PMC6015824

[B29] Friso-Van den Bos I. Van der Ven S. H. G. Kroesbergen E. H. Van Luit J. E. H. (2013). Working memory and mathematics in primary school children: a meta-analysis. *Educ. Res. Rev.* 10 29–44. 10.1016/j.edurev.2013.05.003

[B30] Geary D. C. (2011). Consequences, characteristics, and causes of mathematical learning disabilities and persistent low achievement in mathematics. *J. Dev. Behav. Pediatr.* 32 250–263. 10.1097/DBP.0b013e318209edef 21285895 PMC3131082

[B31] Gertner L. Arend I. Henik A. (2013). Numerical synesthesia is more than just a symbol-induced phenomenon. *Front. Psychol.* 4:860. 10.3389/fpsyg.2013.00860 24348435 PMC3827548

[B32] Glenberg A. M. (2021). Embodiment and learning of abstract concepts (such as algebraic topology and regression to the mean). *Psychol. Res.* 10.1007/s00426-021-01576-5 34468857

[B33] Haig D. (2010). Transfers and transitions: parent–offspring conflict, genomic imprinting, and the evolution of human life history. *Proc. Natl. Acad. Sci.U.S.A.* 107:1731. 10.1073/pnas.0904111106 19666529 PMC2868297

[B34] Heavey L. Hermelin B. Crane L. Pring L. (2012). The structure of savant calendrical knowledge. *Dev. Med. Child Neurol.* 54 507–513. 10.1111/j.1469-8749.2012.04250.x 22409601

[B35] Hilton C. (2019). Fingers matter: the development of strategies for solving arithmetic problems in children with Apert syndrome. *Front. Educ.* 4:131. 10.3389/feduc.2019.00131

[B36] Hutto D. D. Kirchhoff M. D. Abrahamson D. (2015). The enactive roots of STEM: rethinking educational design in mathematics. *Educ. Psychol. Rev.* 27 371–389. 10.1007/s10648-015-9326-2

[B37] Ifrah G. (2001). *The Universal History of Computing: From the Abacus to the Quantum Computer.* New York, NY: John Wiley & Sons Incorporated.

[B38] James J. (1993). *A Calculus of Number Based on Spatial Forms.* Ph.D. thesis. Seattle, WA: University of Washington.

[B39] Jansen B. R. Hofman A. D. Straatemeier M. van Bers B. M. Raijmakers M. E. van der Maas H. L. (2014). The role of pattern recognition in children’s exact enumeration of small numbers. *Br. J. Dev. Psychol.* 32 178–194. 10.1111/bjdp.12032 24862903

[B40] Jonauskaite D. Abu-Akel A. Dael N. Oberfeld D. Abdel-Khalek A. M. Al-Rasheed A. S. (2020). Universal patterns in color-emotion associations are further shaped by linguistic and geographic proximity. *Psychol. Sci.* 31 1245–1260. 10.1177/0956797620948810 32900287

[B41] Katzin N. Cohen Z. Z. Henik A. (2019). If it looks, sounds, or feels like subitizing, is it subitizing? A modulated definition of subitizing. *Psychon. Bull. Rev.* 26 790–797. 10.3758/s13423-018-1556-0 30632105

[B42] Kauffman L. H. (1995). Arithmetic in the form. *Cybernet. Syst.* 26 1–57. 10.1080/01969729508927486

[B43] Kauffman L. H. (2001). The mathematics of Charles Sanders Peirce. *Cybernet. Hum. Know.* 8 79–110.

[B44] Kauffman L. H. Varela F. J. (1980). Form dynamics. *J. Soc. Biol. Struct.* 3 171–206. 10.1016/0140-1750(80)90008-1

[B45] Kaufmann L. Mazzocco M. M. Dowker A. von Aster M. Göbel S. M. Grabner R. H. (2013). Dyscalculia from a developmental and differential perspective. *Front. Psychol.* 4:516. 10.3389/fpsyg.2013.00516 23970870 PMC3748433

[B46] Kline M. (1990). *Mathematical Thought From Ancient to Modern Times: Volume I.* New York, NY: Oxford University Press.

[B47] Knops A. Viarouge A. Dehaene S. (2009). Dynamic representations underlying symbolic and nonsymbolic calculation: evidence from the operational momentum effect. *Attent. Percept. Psychophys.* 71 803–821. 10.3758/APP.71.4.803 19429960

[B48] Kotler J. Haig D. (2018). The tempo of human childhood: a maternal foot on the accelerator, a paternal foot on the brake. *Evol. Anthropol.* 27 80–91. 10.1002/evan.21579 29575348 PMC5947556

[B49] Krajcsi A. Szabó E. Mórocz I. Á (2013). Subitizing is sensitive to the arrangement of objects. *Exp. Psychol.* 60 227–234. 10.1027/1618-3169/a000191 23422657

[B50] Kramer P. Bressan P. (2015). Humans as superorganisms: how microbes, viruses, imprinted genes, and other selfish entities shape our behavior. *Perspect. Psychol. Sci.* 10 464–481. 10.1177/1745691615583131 26177948

[B51] Kramer P. Stoianov I. Umiltà C. Zorzi M. (2011). Interactions between perceptual and numerical space. *Psychon. Bull. Rev.* 18 722–728. 10.3758/s13423-011-0104-y 21562926

[B52] Kucian K. von Aster M. (2015). Developmental dyscalculia. *Eur. J. Pediatr.* 174 1–13. 10.1007/s00431-014-2455-7 25529864

[B53] Laisant C. A. (1915). *Initiation Mathématique: Ouvrage Étranger à Tout Programme, Dédié Aux Amis de L’enfance.* Paris: Hachette & cie.

[B54] Lakoff G. Núñez R. (2000). *Where Mathematics Comes From.* New York, NY: Basic Books.

[B55] Lancy D. F. (1983). *Cross-Cultural Studies in Cognition and Mathematics.* New York, NY: Academic Press.

[B56] Laski E. V. Jor’dan J. R. Daoust C. Murray A. K. (2015). What makes mathematics manipulatives effective? Lessons from cognitive science and Montessori education. *SAGE Open* 5 1–8. 10.1177/2158244015589588

[B57] Liu W. Zheng P. Huang S. Cicchini G. M. (2020). Subitizing, unlike estimation, does not process sets in parallel. *Sci. Rep.* 10:15689. 10.1038/s41598-020-72860-4 32973306 PMC7518424

[B58] Longo M. R. Lourenco S. F. (2010). Bisecting the mental number line in near and far space. *Brain Cogn.* 72 362–367. 10.1016/j.bandc.2009.10.016 19951825

[B59] Lu Y. Li M. Cui Z. Wang L. Hu Y. Zhou X. (2021). Transfer effects of abacus training on cognition. *Curr. Psychol.* 10.1007/s12144-021-01968-1

[B60] Lu Y. Ma M. Chen G. Zhou X. (2020). Adaptive reconfiguration of intrinsic community structure in children with 5-year abacus training. *Psychol. Sch.* 58 235–251. 10.1002/pits.22441 33585902

[B61] Maffia A. Mariotti M. A. (2020). From action to symbols: giving meaning to the symbolic representation of the distributive law in primary school. *Educ. Stud. Math.* 104 25–40. 10.1007/s10649-020-09944-5

[B62] Mareschal D. Butterworth B. Tolmie A. (2013). *Educational Neuroscience.* Chichester: John Wiley & Sons.

[B63] Marghetis T. Núñez R. Bergen B. K. (2014). Doing arithmetic by hand: hand movements during exact arithmetic reveal systematic, dynamic spatial processing. *Q. J. Exp. Psychol.* 67 1579–1596. 10.1080/17470218.2014.897359 25051274

[B64] Matsuzawa T. (2009). Symbolic representation of number in chimpanzees. *Curr. Opin. Neurobiol.* 19 92–98. 10.1016/j.conb.2009.04.007 19447029

[B65] Menon V. Chang H. (2021). Emerging neurodevelopmental perspectives on mathematical learning. *Dev. Rev.* 60:100964. 10.1016/j.dr.2021.100964 34108794 PMC8184018

[B66] Mokkonen M. Koskela E. Procyshyn T. Crespi B. (2018). Socio-reproductive conflicts and the father’s curse dilemma. *Am. Nat.* 192 250–262. 10.1086/698216 30016171

[B67] Moore T. Haig D. (1991). Genomic imprinting in mammalian development: a parental tug-of-war. *Trends Genet.* 7 45–49. 10.1016/0168-9525(91)90230-N 2035190

[B68] Ostrolenk A. Forgeot d’Arc B. Jelenic P. Samson F. Mottron L. (2017). Hyperlexia: systematic review, neurocognitive modelling, and outcome. *Neurosci. Biobehav. Rev.* 79 134–149. 10.1016/j.neubiorev.2017.04.029 28478182

[B69] Patro K. Nuerk H. C. Brugger P. (2018). Visuospatial biases in preschool children: evidence from line bisection in three-dimensional space. *J. Exp. Child Psychol.* 173 16–27. 10.1016/j.jecp.2018.03.002 29649699

[B70] Pia L. Neppi-Modona M. Folegatti A. (2010). Object-centred pseudoneglect for non-verbal visual stimuli. *Exp. Brain Res.* 200 61–66. 10.1007/s00221-009-1954-7 19641909

[B71] Piazza M. Mechelli A. Butterworth B. Price C. J. (2002). Are subitizing and counting implemented as separate or functionally overlapping processes. *Neuroimage* 15 435–446. 10.1006/nimg.2001.0980 11798277

[B72] Proctor R. W. Cho Y. S. (2006). Polarity correspondence: a general principle for performance of speeded binary classification tasks. *Psychol. Bull.* 132 416–442. 10.1037/0033-2909.132.3.416 16719568

[B73] Proverbio A. M. Carminati M. (2019). Finger-counting observation interferes with number processing. *Neuropsychologia* 131 275–284. 10.1016/j.neuropsychologia.2019.06.001 31185228

[B74] Rapin I. (2016). Dyscalculia and the calculating brain. *Pediatr. Neurol.* 61 11–20. 10.1016/j.pediatrneurol.2016.02.007 27515455

[B75] Reani M. Peek N. Jay C. (2019). How different visualizations affect human reasoning about uncertainty: an analysis of visual behaviour. *Comput. Hum. Behav.* 92 55–64. 10.1016/j.chb.2018.10.033

[B76] Roberts D. D. (1973). *The Existential Graphs of Charles S. Peirce.* The Hague: De Gruyter Mouton. 10.1515/9783110226225/pdf

[B77] Schwenk C. Sasanguie D. Kuhn J.-T. Kempe S. Doebler P. Holling H. (2017). (Non-)symbolic magnitude processing in children with mathematical difficulties: a meta-analysis. *Res. Dev. Disabil.* 64 152–167. 10.1016/j.ridd.2017.03.003 28432933

[B78] Shin S. J. (2002). *The Iconic Logic of Peirce’s Graphs.* Cambridge, MA: MIT Press.

[B79] Soylu F. Lester F. K. Jr. Newman S. D. (2018). You can count on your fingers: the role of fingers in early mathematical development. *J. Num. Cogn.* 4 107–135. 10.5964/jnc.v4i1.85 33680184

[B80] Spencer-Brown G. (1969). *Laws of Form.* Leipzig, Germany: Bohmeier Verlag.

[B81] Stoet G. Geary D. C. (2018). The gender-equality paradox in science, technology, engineering, and mathematics education. *Psychol. Sci.* 29 581–593. 10.1177/0956797617741719 29442575

[B82] Stoet G. Geary D. C. (2020). Corrigendum: the gender-equality paradox in science, technology, engineering, and mathematics education. *Psychol. Sci.* 31 110–111. 10.1177/0956797619892892 29442575

[B83] Su R. Rounds J. Armstrong P. I. (2009). Men and things, women and people: a meta-analysis of sex differences in interests. *Psychol. Bull.* 135 859–884. 10.1037/a0017364 19883140

[B84] Szkudlarek E. Zhang H. DeWind N. K. Brannon E. M. (2022). Young children intuitively divide before they recognize the division symbol. *Front. Hum. Neurosci.* 16:752190. 10.3389/fnhum.2022.752190 35280204 PMC8913505

[B85] Thomas N. A. Rose W. Nicholls M. E. R. (2017). The influence of distractors and numerical direction on mental number line bisection. *Laterality* 22 31–48. 10.1080/1357650X.2015.1108329 26529579

[B86] Trafimow D. (2011). Using pictures to enhance students’ understanding of Bayes’ theorem. *Teach. Stat.* 33 83–84. 10.1111/j.1467-9639.2010.00438.x

[B87] Tran C. Smith B. Buschkuehl M. (2017). Support of mathematical thinking through embodied cognition: nondigital and digital approaches. *Cogn. Res.* 2:16. 10.1186/s41235-017-0053-8 28280771 PMC5321706

[B88] Treffert D. A. Christensen D. D. (2005). Inside the mind of a savant. *Sci. Am.* 293 108–113. 10.1038/scientificamerican1205-108 16323698

[B89] Úbeda F. (2008). Evolution of genomic imprinting with biparental care: implications for Prader-Willi and Angelman syndromes. *PLoS Biol.* 6:e208. 10.1371/journal.pbio.0060208 18752349 PMC2525684

[B90] Úbeda F. Gardner A. (2015). Mother and offspring in conflict: why not. *PLoS Biol.* 13:e1002084. 10.1371/journal.pbio.1002084 25785938 PMC4364986

[B91] Ullman M. T. Earle F. S. Walenski M. Janacsek K. (2020). The neurocognition of developmental disorders of language. *Annu. Rev. Psychol.* 71 389–417. 10.1146/annurev-psych-122216-011555 31337273

[B92] Umiltà C. Priftis K. Zorzi M. (2009). The spatial representation of numbers: evidence from neglect and pseudoneglect. *Exp. Brain Res.* 192 561–569. 10.1007/s00221-008-1623-2 18985329

[B93] van den Berg F. C. G. de Weerd P. Jonkman L. M. (2021). Electrophysiological evidence for internalized representations of canonical finger-number gestures and their facilitating effects on adults’ math verification performance. *Sci. Rep.* 11:11776. 10.1038/s41598-021-91303-2 34083708 PMC8175394

[B94] van Leeuwen T. M. Neufeld J. Hughes J. Ward J. (2020). Synaesthesia and autism: different developmental outcomes from overlapping mechanisms. *Cogn. Neuropsychol.* 37 433–449. 10.1080/02643294.2020.1808455 32845799

[B95] van Leeuwen T. M. Wilsson L. Norrman H. N. Dingemanse M. Bölte S. Neufeld J. (2021). Perceptual processing links autism and synesthesia: a co-twin control study. *Cortex* 145 236–249. 10.1016/j.cortex.2021.09.016 34763130

[B96] Wang C. (2020). A review of the effects of abacus training on cognitive functions and neural systems in humans. *Front. Neurosci.* 14:913. 10.3389/fnins.2020.00913 32982681 PMC7492585

[B97] Wang M.-T. Degol J. L. (2017). Gender gap in science, technology, engineering, and mathematics (STEM): Current knowledge, implications for practice, policy, and future directions. *Educ. Psychol. Rev.* 29 119–140. 10.1007/s10648-015-9355-x 28458499 PMC5404748

[B98] Wang Y. (2009). Hands-on mathematics: two cases from ancient Chinese mathematics. *Sci. Educ.* 18 631–640. 10.1007/s11191-007-9078-6

[B99] Winter B. Yoshimi J. (2020). Metaphor and the philosophical implications of embodied mathematics. *Front. Psychol.* 11:569487. 10.3389/fpsyg.2020.569487 33224063 PMC7667247

[B100] Xirocostas Z. A. Everingham S. E. Moles A. T. (2020). The sex with the reduced sex chromosome dies earlier: a comparison across the tree of life. *Biol. Lett.* 16:20190867. 10.1098/rsbl.2019.0867 32126186 PMC7115182

[B101] Zhang Y. Wang C. Yao Y. Zhou C. Chen F. (2021). Adaptive reconfiguration of intrinsic community structure in children with 5-year abacus training. *Cereb. Cortex* 31 3122–3135. 10.1093/cercor/bhab010 33585902

[B102] Zukav G. (1979). *The Dancing Wu Li Masters: An Overview of the New Physics.* New York, NY: Bantam Books.

