# Supplementary material for: Iconic Mathematics: Math Designed to Suit the Mind
Source: Front Psychol. 2022 Jun 13;13:890362. doi: 10.3389/fpsyg.2022.890362 (PMC9234488; doi:10.3389/fpsyg.2022.890362)
Supplement: Supplementary file 1 [file Data_Sheet_1.pdf]

Supplementary Figures

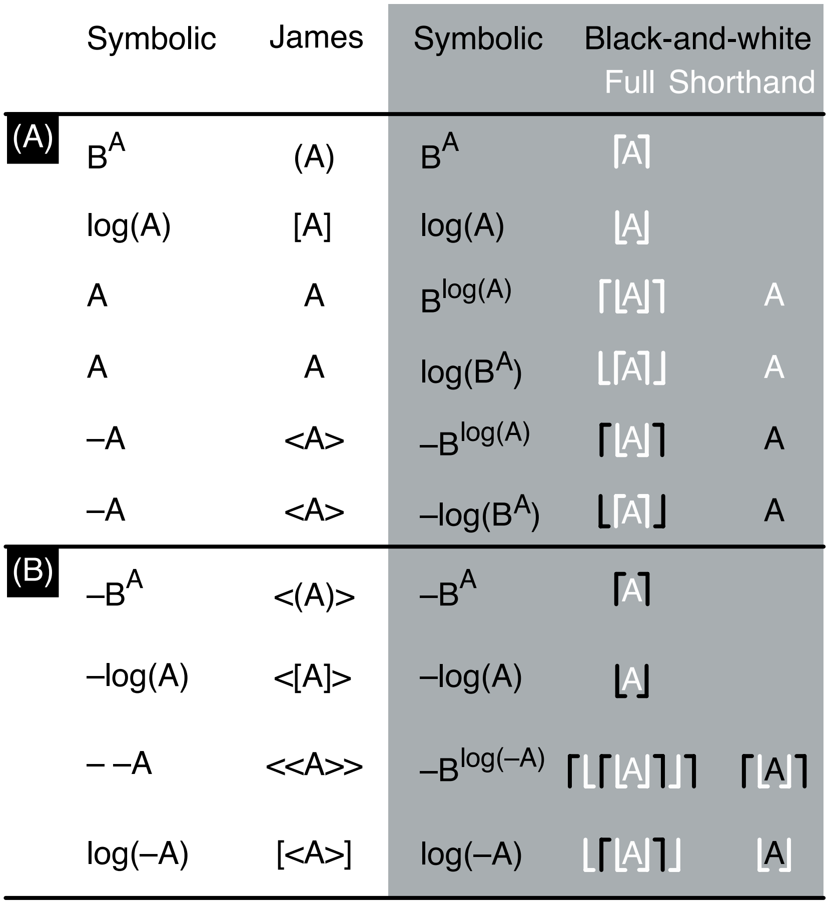

Supplementary Figure 1. A copy of Figure 6 in bracket notation. The bracket notation has the advantage that, as nesting increases, the notation expands horizontally but not vertically. Yet it has the disadvantage that it is less iconic, less clear.

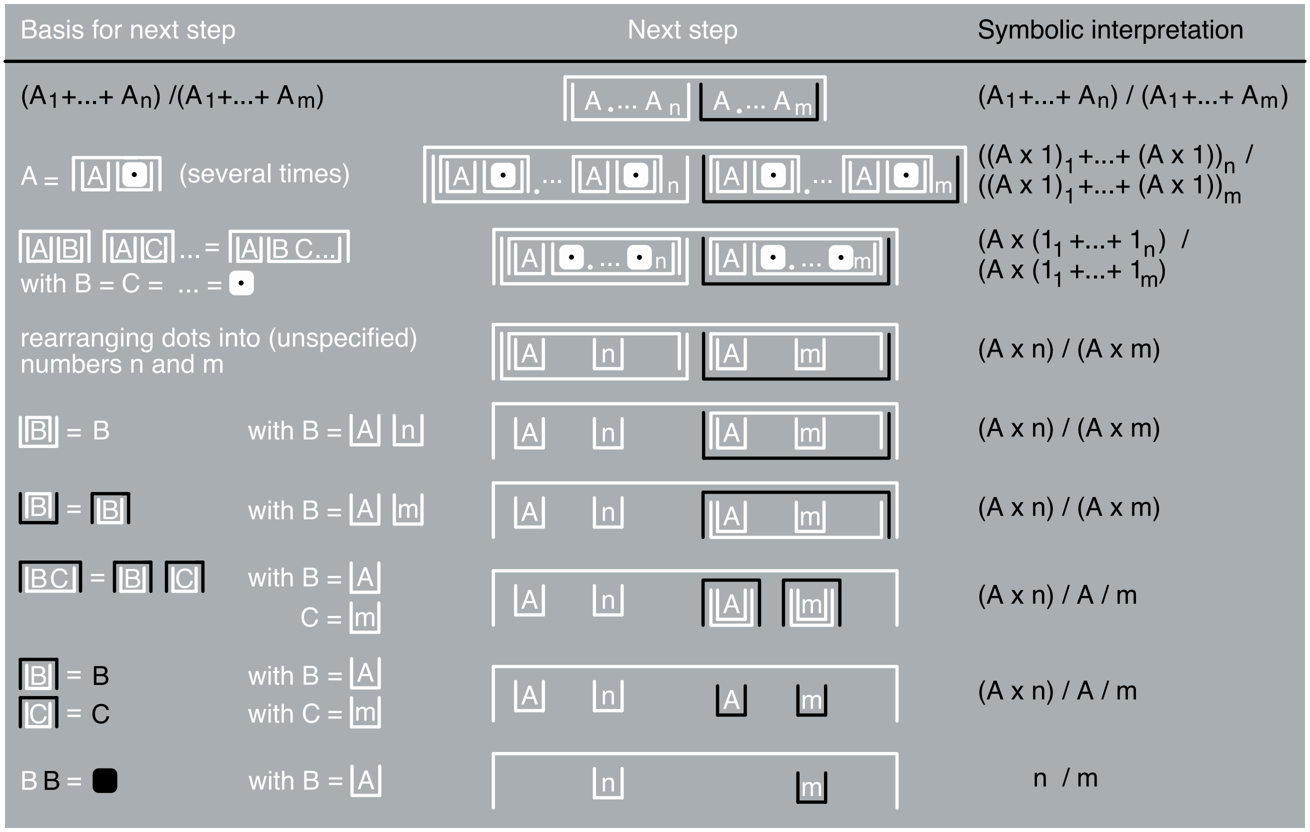

Supplementary Figure 2. Proof of the last additional theorem of Figure 7. The justification of each next step in the derivation is shown (left), the step itself (middle), and a symbolic interpretation in terms of multiplication and division (right). A mathematically equivalent symbolic interpretation in terms of powers and logarithms would be more straightforward but less instructive, and due to symbolic math's rather baroque notation of powers and logarithms (see Figure 7), also considerably harder to read. All logarithms and exponents are assumed to have the same arbitrary base B. Keeping in mind that log(1)=0, the other additional theorems of Figure 7 are considered self-evident and left without proof.

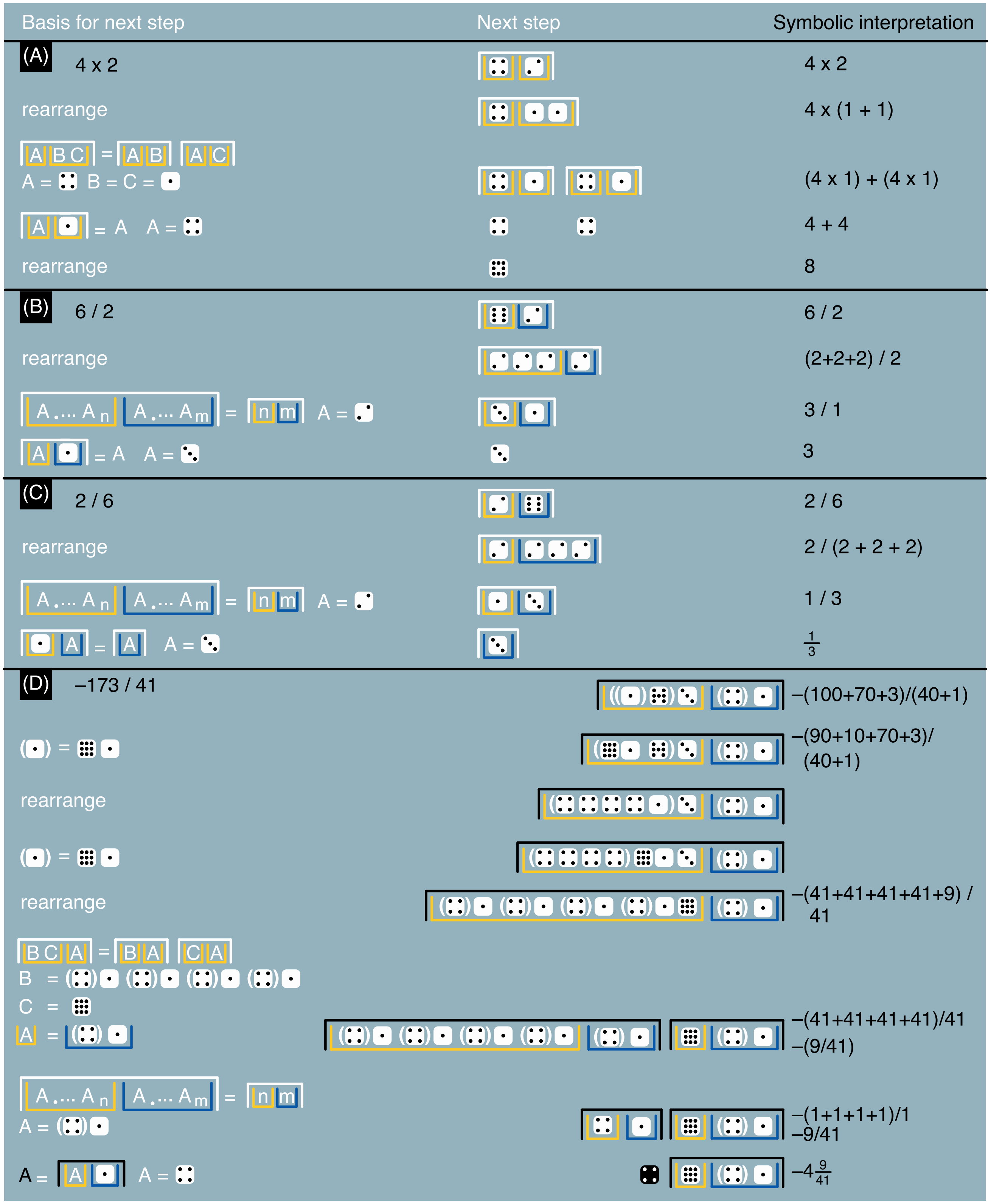

Supplementary Figure 3. A copy of Figure 8 in which, for added clarity, white and black upright containers have been replaced with yellow and blue ones.

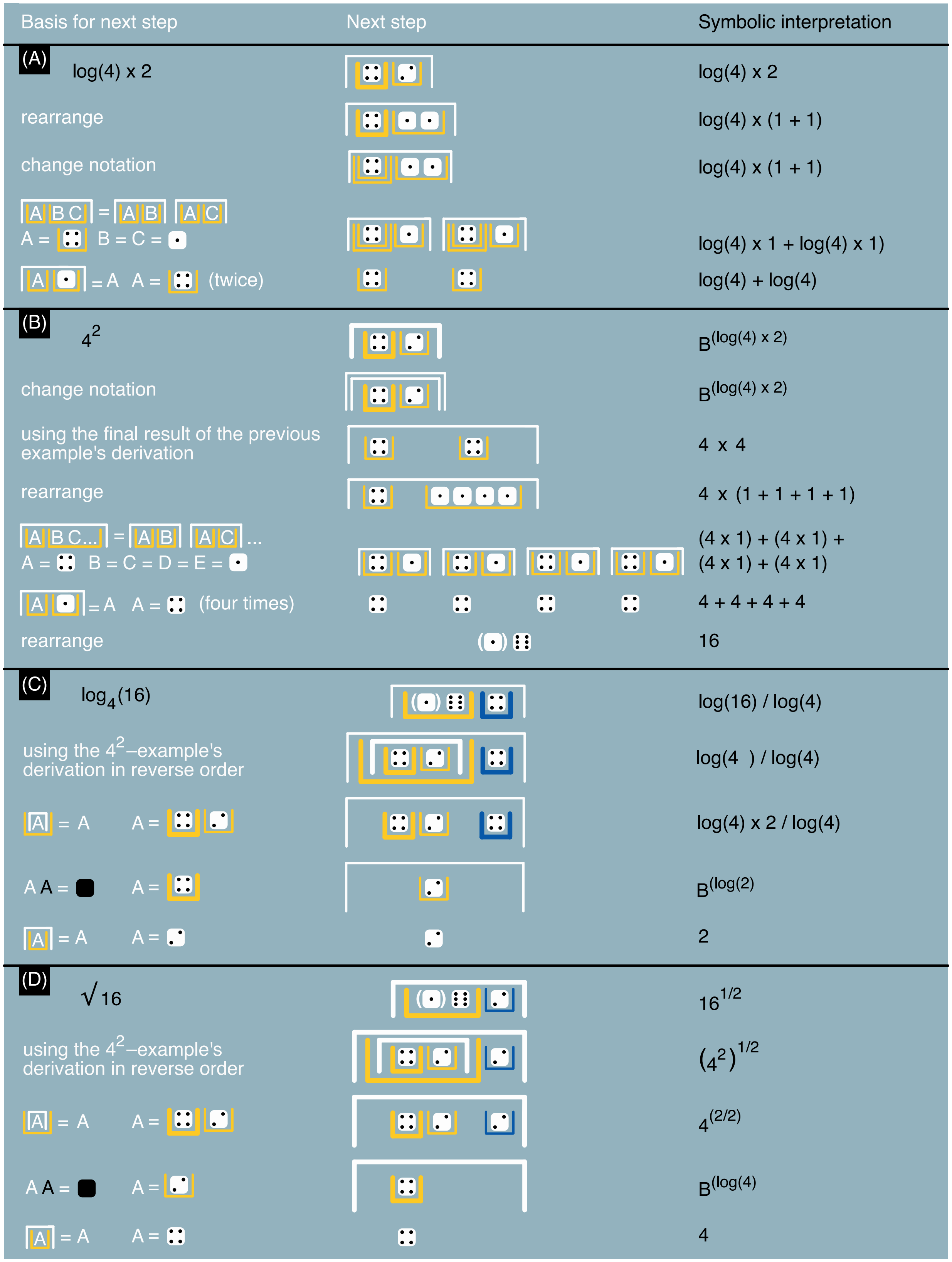

Supplementary Figure 4. A copy of Figure 9 in which, for added clarity, white and black upright containers have been replaced with yellow and blue ones.
